# Delayed fenestration of Blake’s pouch with or without vermian hypoplasia: fetal MRI at 3 tesla versus 1.5 tesla

**DOI:** 10.1186/s40673-019-0098-1

**Published:** 2019-03-05

**Authors:** Thomas Kau, Robert Birnbacher, Peter Schwärzler, Sandra Habernig, Hannes Deutschmann, Eugen Boltshauser

**Affiliations:** 1 Institute of Radiology, Villach General Hospital, Nikolaigasse 43, 9500 Villach, Austria; 2 Department of Pediatrics, Villach General Hospital, Villach, Austria; 3 Department of Gynecology and Obstetrics, Villach General Hospital, Villach, Austria; 4 Institute of Diagnostic and Interventional Radiology, Klinikum Klagenfurt, Klagenfurt, Austria; 5 Division of Neuroradiology, Vascular and Interventional Radiology, Department of Radiology, Medical University of Graz, Graz, Austria; 6 Department of Pediatric Neurology, University Children’s Hospital, University of Zurich, Zurich, Switzerland

**Keywords:** Fetus, Cerebellar vermis, Cranial fossa, posterior, Magnetic resonance imaging, Prenatal diagnosis

## Abstract

**Background:**

Fetal magnetic resonance imaging (MRI), mainly performed at standard field strength, plays a role in the classification of posterior fossa malformations. In the context of early second-trimester screening, upward rotation of the cerebellar vermis per se is usually compatible with a more favorable outcome than Dandy-Walker malformation and profound vermian hypoplasia. Delayed fenestration of Blake’s pouch may either mimic vermian hypoplasia by compression or be associated with it in individual cases. To increase specificity, there is a growing interest in the use of high-field MRI which is believed to be safe as long as the specific absorption rate is kept within accepted limits. We aim to illustrate its added value during the second and third trimester.

**Case presentation:**

In the first case, fetal MRI at 1.5 Tesla was performed at 21 and 27 weeks’ gestation with sonographic follow up postnataly. In the second case, 3 Tesla MR images were acquired at 21 and 34 weeks’ gestation as well as in the neonatal period.

**Conclusions:**

This pictorial case vignette supports the suggestion that mid-gestational MRI at 3 Tesla has the potential to exclude pronounced vermian hypoplasia with higher confidence than at 1.5 Tesla. However, the discrimination of mild hypoplasia from slight deformation of the cerebellar vermis will likely remain challenging.

## Introduction

Mid-sagittal visualisation of the vermis and, therefore, accurate classification of posterior fossa abnormalities as part of the sonographic second-trimester screening can be challenging, making it a frequent indication for fetal magnetic resonance imaging (MRI) [1–3]. While Dandy-Walker malformation and confirmed hypoplasia of the cerebellar vermis correlate with adverse neurologic development in a large proportion of cases, about 90% of fetuses with either isolated Blake’s pouch cyst or mega cisterna magna are reported to have normal developmental outcome [4]. Delayed rotation of the cerebellar vermis may be an imaging pitfall and a potential risk of unnecessary pregnancy interruption [5].

Blake’s pouch cyst is believed to be caused by failed fenestration of the Blake’s pouch, an outpouching of the posterior membraneous area continuous with the fourth ventricle. Embryologically, its neck becomes the foramen of Magendie usually around 18 weeks’ gestation. In cases of persistent Blake’s pouch, the vermis is elevated but the fastigial recess, the primary fissure, and the lobulation appear normal [6]. In contrast to Dandy-Walker malformation, the tegmento-vermian angle will be < 45° in case of a Blake’s pouch cyst or vermian hypoplasia, with an angle of approximately > 25° suggesting the latter rather than the former [7]. A detailed description is to be preferred over unspecific and sometimes confusing terms like “Dandy-Walker variant” or “Dandy-Walker spectrum” [8, 9]. Unfortunately, the term “inferior vermian hypoplasia” is increasingly used despite known limitations in our ability to distinguish it from persistent Blake’s pouch [2, 5, 6]. In his much-noticed editorial on the pre- and misconception of inferior vermian hypoplasia, Robinson pointed out that, since the vermis develops more in a ventral-to-dorsal direction, it may not necessarily be the inferior vermis that is abnormal, or the vermis may not be abnormal at all. [6]. Albeit difficult to detect, the position of the choroid plexus is a potential discriminator between true vermian hypoplasia and Blake’s pouch cyst [6, 10].

In fetal MRI, switching to higher field strength requires adapted imaging sequences, handling of artefacts, and a limitation of the specific absorption rate to 4 W/kg for maternal whole-body exposure [11]. Superior image resolution at 3 Tesla as compared to 1.5 Tesla has recently been proven for a number of brain structures including the cerebellum between 20 and 24 weeks' gestation [12]. Follow-up imaging at a later gestational age most probably further increases diagnostic accuracy. However, spontaneous resolution of Blake’s pouch in the third trimester will not necessarily answer the question if the vermis is normal or not.

Here, we aim to illustrate the potential of second- and third-trimester MRI at 3 Tesla and assess its added value in persistent Blake’s pouch with suspected vermian hypoplasia.

## Pictorial case vignette

### Case 1

**Fig. 1:**
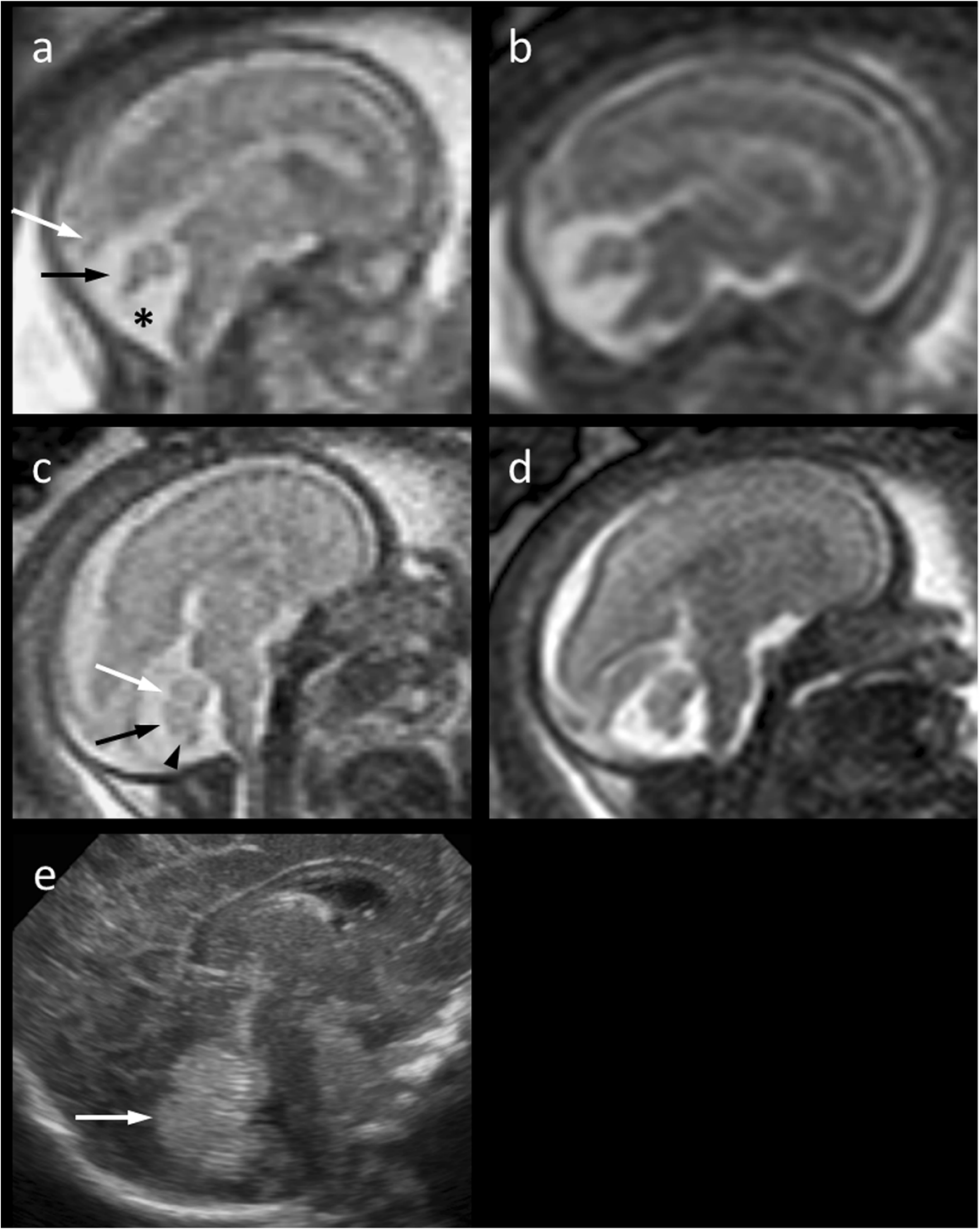
**a–d** Mid-sagittal T2-weighted 1.5 Tesla magnetic resonance images (**a** and **c**, Single-shot; **b** and **d**, Balanced Turbo Field Echo) of the fetal brain. At 21 weeks‘gestation (**a**, **b**), the cerebellar vermis (**a**, *black arrow*) appears upwardly rotated and moderately hypoplastic with a normal torcular position (**a**, *white arrow*). At 27 weeks‘gestation (**c**, **d**), the vermis appears nearly normal in position, shape and size suggesting delayed fenestration of Blake’s pouch (**a**, *asterisk*) The primary (**c**, *white arrow*), prepyramidal (**c**, *black arrow*), and secondary (**c**, *arrow head*) fissures are roughly discernible. **e** A slight uncertainty regarding minimal hypoplasia of the cerebellar vermis (*arrow*) remains even after inconspicuous transcranial ultrasound postnatally at the age of 10 weeks

A 29 year-old woman was referred for fetal MRI at 21 weeks’ gestation because of suspected Dandy-Walker malformation according to mid-gestational ultrasound. Common chromosomal anomalies had been excluded. Magnetic resonance imaging using a Phillips 1.5 Tesla scanner revealed moderate rotation of the cerebellar vermis (Fig. 1a, b) which we believed was due to mild vermian hypoplasia. Dandy-Walker malformation was, therefore, excluded. Follow-up MRI at 1.5 Tesla was performed at 27 weeks’ gestation. Rotation of the vermis was less pronounced at that time and, given a cross-sectional area of 103 mm^2^ on a mid-sagittal single-shot T2-weighted image, vermian hypoplasia was rated minimal if present at all (Fig. 1c, d). Cesarean section was performed at 28 weeks’ gestation due to premature rupture of membranes after amniocentesis with subsequent intra-amniotic infection symptoms. In postnatal transcranial ultrasound at the age of 10 weeks, the cerebellar vermis appeared normal (Fig. 1e). Following intensive care for infant respiratory distress syndrome, the girl developed normally and was neurologically unremarkable at the corrected age of 3 ¾ years.

### Case 2

**Fig. 2:**
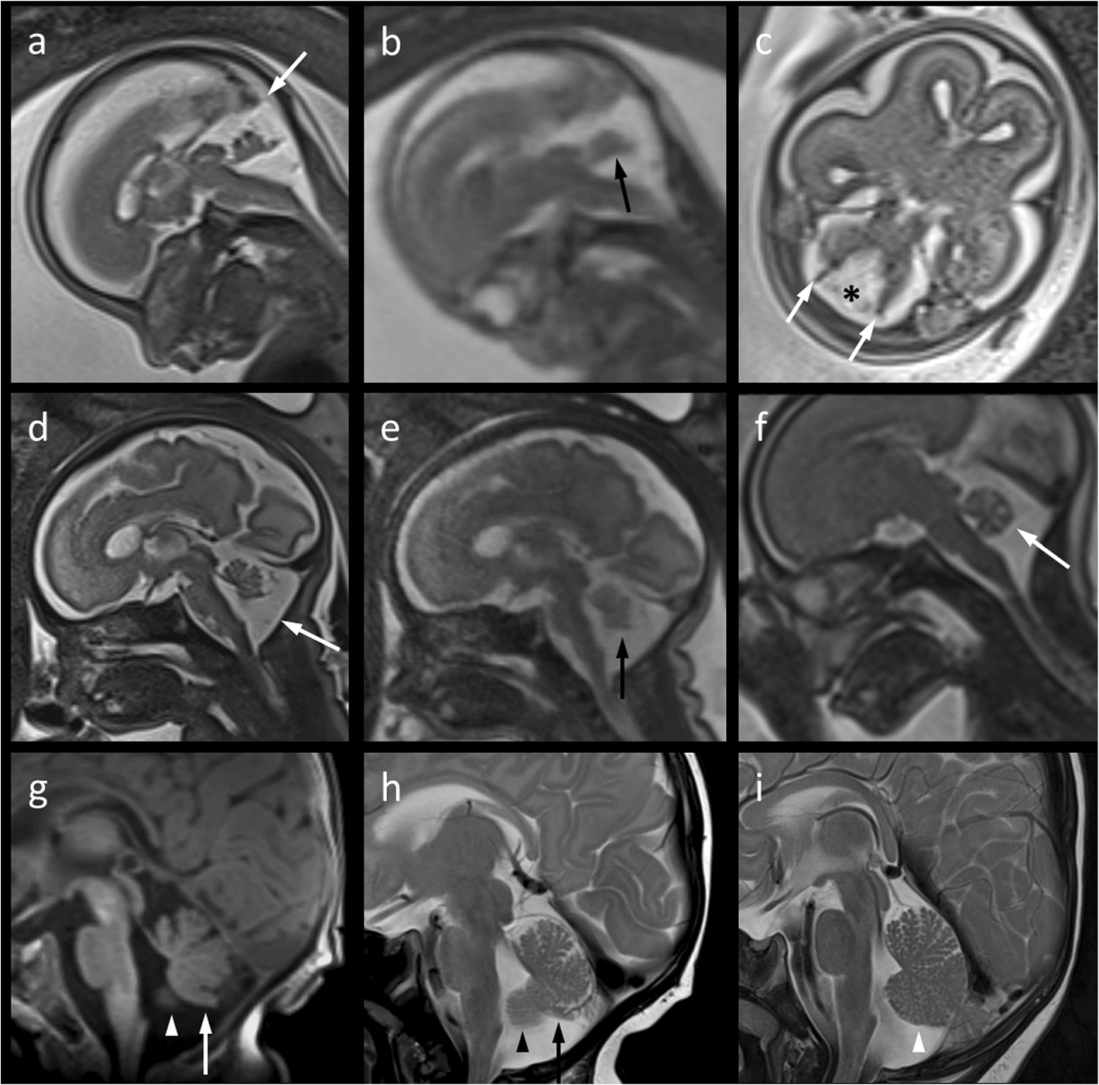
**a–c** Mid-sagittal (**a**, Half-Fourier Acquisition Single-Shot Turbo Spin-Echo [HASTE]; **b**, True Fast Imaging With Steady-State Free Precession) and axial (**c**, HASTE) T2-weighted 3 Tesla magnetic resonance (MR) images of the fetal brain at 21 weeks‘gestation suggesting a moderately hypoplastic cerebellar vermis with a flattened fastigial point (**b**, *arrow*) and moderately increased tegmento-vermian angle of about 35°, but normal torcular position (**a**, *arrow*). Lateral septa (**c**, *arrows*) in the posterior fossa are believed to belong to Blake’s pouch (**c**, *asterisk*). **d**, **e** Follow-up imaging on the same scanner at 31 weeks‘gestation shows nearly normal rotation of the vermis in a slightly enlarged posterior fossa (**d**, *arrow*) suggesting delayed fenestration of Blake’s pouch. Mild infero-posterior vermian hypoplasia (**e**, *arrow*) may be suspected from early third-gestational MR imaging (MRI) (**d**, **e**) This finding becomes more evident if compared to the sagittal T2 HASTE image of the cerebellar vermis (**f**, *arrow*) in a fetus at 24 weeks‘gestation scanned for suspected pulomonary sequestration on the same MR system. **g**, **h** A similar pattern is depicted by postnatal sagittal MRI (**g**, T1 Magnetization-Prepared Rapid Gradient-Echo; **h**, T2 Turbo spin echo) at the age of 12 weeks. Size and shape of the cerebellar vermis imply that it is mildly hypoplastic and its posterior lobe has experienced mass effect due to prolonged persistence of Blake’s pouch. Further, partial volume of cerebellar hemisphere (**g**, **h**; *arrow heads*) adjacent to the foramen of Magendie has to be considered. **i** For comparison of cerebellar volume and fissuration, the sagittal T1-weighted MR image in a 4-month-old, clinically inapparent infant scanned for a temporopolar arachnoid cyst – known from pre- and postnatal ultrasound – is given here

A 33 year-old woman was referred for fetal MRI at 21 weeks’ gestation for clarification of a suspected malformation in the posterior fossa. The following differential diagnostic suggestions were given after mid-gestational ultrasound: *Mega cisterna magna, Blake’s pouch, Dandy-Walker sequence*? Fetal imaging was performed on a 3 Tesla Siemens Magnetom Vida scanner. On MRI, the infero-posterior part of the cerebellar vermis appeared to be moderately hypoplastic (Fig. 2a, b). This was associated with a tegmento-vermian angle of 35 degrees, most probably due to non-perforation of Blake’s pouch. Dandy-Walker malformation (in the narrow sense) could be excluded (Fig. 2a). Follow-up images acquired on the same scanner at 31 weeks’ gestation depicted a slightly pronounced cisterna magna and a nearly normalized tegmento-vermian angle (Fig. 2d, e). The cross-sectional area of the vermis on a mid-sagittal Half-Fourier Acquisition Single-Shot Turbo Spin-Echo (HASTE) image was 112 mm^2^. With this pattern, we were unsure if the vermis was slightly hypoplastic or only compressed inferiorly as a consequence of delayed perforation of Blake’s pouch. Showing a very similar imaging pattern, 3 Tesla MRI at the age of 12 weeks confirmed the fetal imaging report, but did not add any relevant information (Fig. 2g, h). The boy was neurologically unremarkable at the age of 3 months.

## Discussion

In a report of two cases, Pinto et al. pointed out that incomplete rotation of the cerebellar vermis could be a physiological finding in the early second trimester compatible with normal neurological development [5]. The authors questioned the diagnostic specificity of fetal 1.5 Tesla MRI in the accurate distinction of severe from prognostically less relevant malformations of the posterior fossa at that stage. Further, they recommended follow-up at a later gestational age. Potentially mimicking Dandy-Walker malformation or marked vermian hypoplasia, persistent Blake’s pouch – used interchangeably with Blake’s pouch cyst – may even account for unjustified interruptions of pregnancy [4, 5].

With the present pictorial review of two cases, we aim to illustrate the potential of fetal MRI at both 1.5 and 3 Tesla including postnatal correlation. Reflecting the current literature, four aspects deserve special attention and we would like to discuss them stimulated by four questions:

1. *Will improved image resolution translate into higher diagnostic confidence with fetal MRI at 3 Tesla?* In general, the signal-to-noise ratio at 3 Tesla is nearly two times as high as with 1.5 Tesla, which can either be used to improve image quality or to reduce scan time [13, 14]. This potential benefit of high-field MRI has also been proven for imaging the cerebellum in the late second trimester despite the challenge of additional artefacts [12]. In our own experience, fetal MRI at 3 Tesla enabled us to exclude more severe vermian hypoplasia with higher confidence already at 21 weeks‘gestation, thus, following ultrasound screening. At 31 weeks‘gestation, the image pattern of possible mild vermian hypoplasia on T2-HASTE images may already resemble that of postnatal MRI (Fig. 2d, g, h).
2. *Is follow-up imaging the key to an accurate antenatal assessment of the cerebellar vermis?* With higher confidence at 3 Tesla compared to 1.5 Tesla, Dandy-Walker malformation and severe vermian hypoplasia could already be excluded at 21 weeks‘gestation in both cases demonstrated here. In accordance with a previously published case [5], we assumed a cystic structure from lateral laminae in the cisterna magna (Fig. 2c). Follow-up MRI, as recommended by Pinto et al. [5], finally confirmed a Blake’s pouch to be responsible for delayed vermian rotation by showing near-normalization of the tegmento-vermian angle at 27 and 31 weeks‘gestation, respectively (Figs. 1 and 2). Still, we were not quite sure about the presence of mild vermian hypoplasia in the context of prolonged persistence of Blake’s pouch. However, since it is the most dorsal part of the vermis that is last to differentiate, elevation of the vermis by the persistent Blake’s pouch will most probably compress the inferior vermis rather than it being hypoplastic [6].
3. *If delayed fenestration of Blake’s pouch is the most likely diagnosis based on imaging follow-up, will vermian hypoplasia rather be mimicked or an associated finding?* The popularity of the prenatal diagnosis of Blake’s pouch cyst has grown with image quality. This term is used for a posterior fossa cyst displacing superiorly an otherwise normal cerebellar vermis, typically without ventricular dilatation [4]. In their metaanalysis of isolated posterior fossa malformations, D’Antonio et al. hypothesized that many false positive cases of isolated vermian hypoplasia in fetal MRI would explain a surprisingly high rate of favorable outcomes [15]. In this context, we would like to draw attention to a possible difference between our second case (Fig. 2) on the one hand and the two cases published by Pinto et al. [5] as well as – with some uncertainty – our first case (Fig. 1). Specifically, delayed fenestration of Blake’s pouch may not invariably mimic mild vermian hypoplasia but rather coincide or be associated with it. Persistence of Blake’s pouch causing elevation of an intrinsically normal vermis and deficiency in any part of the vermis can both give the appearance of inferior vermian hypoplasia [6]. Notably, vermian hypoplasia may not necessarily affect or be restricted to its inferior part [6]. Visually compared to normal controls (e.g., Fig. 2i), the cerebellar vermis in our second case remained suspicious of mild hypoplasia in addition to slight deformation even after postnatal MRI follow-up (Fig. 2g, h). Hypoplasia was supported by semi-automatic measurement of the vermian cross-sectional area with reference to biometric data published by Ber et al. [16]. According to this publication which suffers from low data density between 25 and 27 weeks‘gestation, vermian cross-sectional area in our first case was normal at the time of follow-up [16].
4. *What is the clinical impact of confirming or excluding mild vermian hypoplasia and how confident can we be?* About 90% of fetuses with isolated Blake’s pouch cyst or mega cisterna magna can be expected to develop normally compared with only 50% of those affected by Dandy-Walker malformation and about 50–75% of those with isolated vermian hypoplasia [4, 17]. Only one out of twenty infants with a prenatal diagnosis of Blake’s pouch cyst showed mild psychomotor disorder at 1–5 years in a study by Gandolfi Colleoni [4]. A few aspects potentially influence such numbers and the individual prognosis: First, vermian hypoplasia may have been over-diagnosed at least in fetal MRI at 1.5 Tesla and ultrasound [5, 15]; second, the suggestion of *mild* vermian hypoplasia will likely become more frequent with the increased use of high-field MR machines; third, despite being less frequent, vermian hypoplasia may be associated with prognostically more critical malformations still challenging for fetal imaging. Finally, our experience supports a generally favourable – if not normal – outcome in suspected (or doubtful) mild vermian hypoplasia.

## Conclusion

Delayed fenestration of Blake’s pouch may be associated with vermian hypoplasia, mimic or coincide with it in individual cases. This pictorial case vignette supports the suggestion that mid-gestational MRI at 3 Tesla has the potential to exclude pronounced vermian hypoplasia with higher confidence than at 1.5 Tesla. Follow-up in the early third trimester is recommended to confirm persistent Blake’s pouch to be responsible for delayed vermian rotation. However, based on a limited level of evidence, the discrimination of mild hypoplasia from slight deformation of the cerebellar vermis remains challenging.

## Abbreviations

HASTE: Half-Fourier Acquisition Single-Shot Turbo Spin-Echo
MRI: Magnetic resonance imaging

## References

[1] Limperopoulos C, Robertson RL, Estroff JA, Barnewolt C, Levine D, Bassan H (2006). Diagnosis of inferior vermian hypoplasia by fetal magnetic resonance imaging: potential pitfalls and neurodevelopmental outcome. Am J Obstet Gynecol.

[2] D'Antonio F, Khalil A, Garel C, Pilu G, Rizzo G, Lerman-Sagie T (2016). Systematic review and meta-analysis of isolated posterior fossa malformations on prenatal ultrasound imaging (part 1): nomenclature, diagnostic accuracy and associated anomalies. Ultrasound Obstet Gynecol.

[3] Katorza E, Bertucci E, Perlman S, Taschini S, Ber R, Gilboa Y (2016). Development of the fetal vermis: new biometry reference data and comparison of 3 diagnostic modalities-3D ultrasound, 2D ultrasound, and MR imaging. AJNR Am J Neuroradiol.

[4] Gandolfi Colleoni G, Contro E, Carletti A, Ghi T, Campobasso G, Rembouskos G (2012). Prenatal diagnosis and outcome of fetal posterior fossa fluid collections. Ultrasound Obstet Gynecol.

[5] Pinto J, Paladini D, Severino M, Morana G, Pais R, Martinetti C (2016). Delayed rotation of the cerebellar vermis: a pitfall in early second-trimester fetal magnetic resonance imaging. Ultrasound Obstet Gynecol.

[6] Robinson AJ (2014). Inferior vermian hypoplasia – preconception, misconception. Ultrasound Obstet Gynecol.

[7] Volpe P, Contro E, De Musso F, Ghi T, Farina A, Tempesta A (2012). Brainstem-vermis and brainstem-tentorium angles allow accurate categorization of fetal upward rotation of cerebellar vermis. Ultrasound Obstet Gynecol.

[8] Bosemani T, Orman G, Boltshauser E, Tekes A, Huisman TA, Poretti A (2015). Congenital abnormalities of the posterior fossa. Radiographics..

[9] Poretti A, Boltshauser E, Huisman TA (2016). Pre- and postnatal neuroimaging of congenital cerebellar abnormalities. Cerebellum..

[10] Nelson MD, Maher K, Gilles FH (2004). A different approach to cysts of the posterior fossa. Pediatr Radiol.

[11] Weisstanner C, Gruber GM, Brugger PC, Mitter C, Diogo MC, Kasprian G (2017). Fetal MRI at 3T – ready for routine use?. Br J Radiol.

[12] Priego G, Barrowman NJ, Hurteau-Miller J, Miller E (2017). Does 3T fetal MRI improve image resolution of Normal brain structures between 20 and 24 Weeks’ gestational age?. AJNR Am J Neuroradiol.

[13] Victoria T, Jaramillo D, Roberts TP, Zarnow D, Johnson AM, Delgado J (2014). Fetal magnetic resonance imaging: jumping from 1.5 to 3 tesla (preliminary experience). Pediatr Radiol.

[14] Victoria T, Johnson AM, Edgar JC, Zarnow DM, Vossough A, Jaramillo D (2016). Comparison between 1.5-T and 3-T MRI for fetal imaging: is there an advantage to imaging with a higher field strength?. AJR Am J Roentgenol.

[15] D'Antonio F, Khalil A, Garel C, Pilu G, Rizzo G, Lerman-Sagie T (2016). Systematic review and meta-analysis of isolated posterior fossa malformations on prenatal imaging (part 2): neurodevelopmental outcome. Ultrasound Obstet Gynecol.

[16] Ber R, Bar-Yosef O, Hoffmann C, Shashar D, Achiron R, Katorza E (2015). Normal fetal posterior Fossa in MR imaging: new biometric data and possible clinical significance. AJNR Am J Neuroradiol.

[17] Tarui T, Limperopoulos C, Sullivan NR, Robertson RL, du Plessis AJ (2014). Long-term developmental outcome of children with a fetal diagnosis of isolated inferior vermian hypoplasia. Arch Dis Child Fetal Neonatal Ed.

